# Investigation of soils affected by burnt hospital wastes in Nigeria using PIXE

**DOI:** 10.1186/2193-1801-2-208

**Published:** 2013-05-07

**Authors:** Inyang Ephraim P, Akpan Ita, Obiajunwa Eusebius I

**Affiliations:** Department of Physics, University of Calabar, Calabar, Nigeria; Centre for Energy Research and Development, Obafemi Awolowo University, Ile Ife, Nigeria

**Keywords:** PIXE, Burnt hospital waste, Hazardous waste, Chlorine, Lead

## Abstract

Improper management of hospital waste has been reported to be responsible for several acute outbreaks like the severe acute respiratory syndrome (SARS). In spite of these challenges, hospital wastes are sometimes not properly handled in Nigeria. To date, there has not been an adequate study on the effect and fate of burnt hospital waste on agricultural soil. The effect of burnt hospital wastes on the agricultural soil was conducted on soils sampled around farm settlement near Obafemi Awolowo University Teaching Hospital Complex, Ile-Ife, South West Nigeria. PIXE technique was employed with a 1.7 MV 5SDH Tandem Pelletron accelerator available at Centre for Energy Research and Development O.A.U Ile-Ife, Nigeria. Eleven elements- Si, Cl, K, Ca, Ti, V, Cr, Mn, Fe, Zr and Pb were detected and their concentrations and enrichment factors determined. The presence of Pb and Cl at the elevated concentrations range of (77.8 ± 3.5 - 279.6 ± 97.6 and 102.2 ± 37.4 -167.2±17.43) ppm respectively in this study, is of serious health concern because of the agricultural practices in the neighborhoods of the study sites. There is a need for proper handling of hospital and other related hazardous wastes because of the possibility of such posing serious environmental pollution problems.

## Introduction

Hospital wastes are generated as a result of patients’ diagnosis and/or treatment or immunization of human beings or animals. Hospital wastes are a universal set having subsets like infectious and hazardous wastes.

Wrongly managed hospitals wastes can result in severe health hazards. It has been reported that hospital waste is one of the most toxic Waste, (see http://www.epa.gov/waste). Countries with little or no proper hospital waste management are prune to severe chronic respiratory syndrome (SARS). Several accidents have been reported where mishandling of hospital wastes led to infections (Shang and Jia, 2002). Hospital wastes are so infectious/hazardous that every means of improper disposal pose a threat to the environments. Studies so far in Nigeria have revealed a zero level of proper management of hospital wastes, in spite of the risk associated with this knowledge gap (Abah and Ohimain (2011; Ngwuluka et al. 2009.

With the ever increasing population of Nigerians, there is a corresponding increase in health care delivery/facilities; the amount of hospital waste generated is also increasing substantially. About 2.5 million tons of waste is generated per year around OAUTHC complex (Inyang, 2010). A large amount of solid waste is generated in the hospital during diagnosis and treatment of diseases. The solid waste may contain human organs, bandages, syringes, test tubes, tissues cell culture and other plastic materials. These wastes can cause health hazards and indeed, is a veritable source of transmission of HIV/AIDS, Hepatitis B and other diseases. The incinerator is an effective and hygienic way for disposal of hospital waste. It is only in very few teaching hospitals in Nigeria that there exits functional incinerators. In Western Europe more than 600 incinerator plants are in operation Li et al., 2003. The dumping of infectious/toxic waste on the soil and sometimes burning them like the case in this study, can lead to contamination of crops and underground water which can cause very serious harm to unsuspected consumers.

Soil is a very specific component of the biosphere because it is not only a geochemical sink for contaminants, but also acts as natural buffer controlling the transport of chemical elements and substances to the atmosphere, hydrosphere and biota. However, the most important role of soil is its productivity, which is the basis for the survival humans (Kabata-Pendias and Pendias 2001).

Hospital solid waste has been found to contain appreciable quantity of heavy metals such as Cd, Zn, Pb and Cu, all which may eventually end-up in the soil and leached down the profile (Shang and Jia (2002). The concern about heavy metals is that they are not biodegradable and therefore accumulate in the environment. Thus one of the development challenges facing this decade is how to achieve a cost effective and environmentally sound strategies to deal with the global hazardous waste crises facing both the developed and developing Countries (Kabata-Pendias and Pendias 2001; Parker and Corbilt 1992; Jensen (1990; NEST 1991; Oyediran AB 1994; Alloway and Ayres 1997).

The present study, which is preliminary, was conducted with an objective to determine the effect of burnt hospital waste on the soil total elemental compositions around the dump sites of the Obafemi Awolowo University Teaching Hospital Complex in Ile – Ife. There appear to be various agricultural practices at the site of this study and around the neighborhoods. Investigation of this kind was very imperative owing to the absence of this type of study in literature to the best of our knowledge.

## Materials and methods

### Study area

The study area is around Obafemi Awolowo University Teaching Hospital Complex, located in Ife East Local Government area of Osun State in South West Nigeria, which lies between latitudes 7°27' and 7° 32' and longitudes 4° 22' and 4° 29'. The geology of the area forms a complex pattern of coarse and fined grained gneisses. The soil is derived from material of the old basement complex, which is mainly made up of granitic metamorphosed sedimentary rock.

### Soil sampling and pelleting

Soil samples were collected from four burnt hospital waste dump sites and a forest site opposite the dump sites as the control. Each sample site was divided into four quadrants each 3 m^2^; and a total of 5 cores soil per quadrant were collected from three of the four quadrants randomly at the depth of 0–15 cm using a stainless steel Dutch auger in composite replicate. The soil samples were thoroughly mixed in clean plastic buckets before sub samples were collected, taken to the laboratory, air dried and sieved through 2 mm sieve. The dried samples for pelleting were ground in agate mortar/pestle. A 20% ultrapure Carbon was added to each of the samples. The soil sample and the ultrapure carbon where afterward homogenised in the agate mortar before pelletization was done. The protocol adopted so as to avoid the cross contamination of the samples during grinding and pelleting are detailed somewhere Inyang et al., and a total of 5 cores soil per quadrant were collected from three of the four quadrants randomly at the depth of 0–15 cm using a stainless steel Dutch auger in composite replicate. The soil samples were thoroughly mixed in clean plastic buckets before sub samples were collected, taken to the laboratory, air dried and sieved through 2 mm sieve. The dried samples for pelleting were ground in agate mortar/pestle. A 20% ultrapure Carbon was added to each of the samples. The soil sample and the ultrapure carbon where afterward homogenised in the agate mortar before pelletization was done. The protocol adopted so as to avoid the cross contamination of the samples during grinding and pelleting are detailed somewhere Inyang et al., and a total of 5 cores soil per quadrant were collected from three of the four quadrants randomly at the depth of 0–15 cm using a stainless steel Dutch auger in composite replicate. The soil samples were thoroughly mixed in clean plastic buckets before sub samples were collected, taken to the laboratory, air dried and sieved through 2 mm sieve. The dried samples for pelleting were ground in agate mortar/pestle. A 20% ultrapure Carbon was added to each of the samples. The soil sample and the ultrapure carbon where afterward homogenised in the agate mortar before pelletization was done. The protocol adopted so as to avoid the cross contamination of the samples during grinding and pelleting are detailed somewhere Inyang et al., 2012. From each sample 13 mm diameter of about 20 g weight thick pellets were made with Spec-caps by applying 10 t pressure with hydraulic pelletized machine. The soil sampling was conducted in the month of July, 2010, about the peak of rain season when topsoil is predominantly wet with fresh loading of metal contaminants.

The International Atomic Energy Agency (IAEA) standard soil 7 was equally pressed in similar manner as samples and these were used for quality assurance.

### The PIXE experiment

Proton induced X-ray emission (PIXE) experiment was performed using a 2.5 MeV proton beam obtained from the 1.7 MV tandem pelletron accelerator (model 5SDH) at the centre for Energy Research and Development, Obafemi Awolowo University Ile-Ife, Nigeria. The measurements were carried with the beam sport of 4 mm in diameter and a beam current of 0.2 to 0.7 nA. The irradiation was for about 158 to 560 s. A Canberra Si(Li) detector Model ESLX 30–150, with the associated pulse processing electronics and Canberra Genie 2000 (3.1) MCA card interfaced to a PC were used for the X-ray data acquisition. With respect to the beam direction, the sample’s normal was located at 0^o^ and the Si(Li) detector at 45°. The PIXE spectra were analyzed using GUPIXWIN version 2.1 program software. To calibrate the PIXE system, the H-value method was used for spectra from thick standards which were measured and compared (within 5%).

## Results and discussion

One of the main goals of this study was to ascertain whether burnt hospital waste has any effect on the total elemental composition of soil. This was very necessary because there appear to be an extensive farming at the site and the surrounding of the study area. Our data reveal an elevated concentration and even the presence anthropogenic elements when compared with the control.

The elemental compositions of the four sites studied are given in Table 1. Eleven elements (Si, Cl, K, Ca, Ti, V, Cr, Mn, Fe, Zr, and Pb) were detected and their concentration determined. As can be seen from Table 1; the dominant trend is as follows: Si > Fe > Ca > K > Ti > Zr > Cl > Cr > V > Pb > Mn. The values obtained in this study which are above the maximum allowable contents of metals for agricultural purposes as proposed by Blankenship et al. , the dominant trend is as follows: Si > Fe > Ca > K > Ti > Zr > Cl > Cr > V > Pb > Mn. The values obtained in this study which are above the maximum allowable contents of metals for agricultural purposes as proposed by Blankenship et al. the dominant trend is as follows: Si > Fe > Ca > K > Ti > Zr > Cl > Cr > V > Pb > Mn. The values obtained in this study which are above the maximum allowable contents of metals for agricultural purposes as proposed by Blankenship et al. 1994in these soils are indicative of anthropogenic action of the burnt hospital waste dump on the soil total elemental composition. For example, the mean content of Zn obtained by other investigators with soil of anthropogenic sources in different countries ranged from 17 to 125 ppm (Blankenship et al. 1994; Kramlich et al. 1989) which is in agreement with the findings in this study. The lead concentration in the top horizon of soil contaminated with anthropogenic activities in other places with similar pedon ranged is about 100 to 189 ppm (EPA 1985; EPA 1987a) this is in conformity with the results obtained in this investigation.

**Table 1:** **PIXE results for soil samples around burnt Hospital waste, the measurements was done with 2.5 MeV protons**

ELEMENTS	CONCENTRATION ppm				
	SITE 1	SITE 2	SITE 3	SITE 4	CONTROL
Si	11.6%±0.51%	13.9%±0.60%	23.1%±0.84%	27.9%±0.85%	18.3%±0.65%
Cl	279.6±29.76	163.1±33.67	136.87±41.56	77.8±13.54	ND
K	1704.2±16.93	1864±16.76	2341.5±16.74	6883.6±15.72	7935.4±19.81
Ca	3440.7±18.54	4328.6±17.33	4110.8±21.36	4562.3±57.23	3514.7±71.09
Ti	2837.5±76.54	3482.2±77.62	2954.1±85.85	4834.0±68.72	ND
V	151.4±24.51	172.7±11.32	166.5±34.34	162.7±26.55	ND
Cr	232.4±14.32	ND	211.5±15.32	117.3±29.82	960.5±11.51
Mn	72.7±1.32	90.0±13.34	330.1±14.52	227.4±6.41	920.9±41.96
Fe	54210.0±43.2	65085.2±54.3	78201±65.4	81228.8±54.9	29110±76.9
Zr	971±7.10	273.7±15.91	177.8±22.72	1055.0±66.94	1004.1±87.21
Pb	119.9±26.96	102.2±29.87	167.2±17.43	102.2±37.43	ND

*ND* = Not Detected.

*Errors* = Counting Statistics.

PIXE results for soil samples around burnt Hospital waste, the measurements was done with 2.5 MeV protons. Concentrations of elements are given in ppm except for major elements which was given in percentage.

Interestingly, the presence of elevated concentrations Cl and Pb in the sites of burnt hospital waste dump and the absent of the same in the control site are of serious environmental and health concern owing to fact that Lead is very toxic elements and Chlorine in particular is related to dioxin and furans emission in the environment. Emphatically Cl with half life () transfer from soil to plant has been reported to be very high (Kashparov et al. 2007). Chlorine has been proven to be an active ingredient essential for dioxin formation during combustion of PVC related material which is a major constituent of hospital related waste materials (EPA 1987b; EPA 2000; Lemiuex 1997; Carrol et al., 1996). The exposure to dioxins and furans can result in liver, kidney or lungs damage. Dioxins and furans are also known as human carcinogen (Carrol et al., 1996). This became very worrisome as there appear to be extensive subsistence agricultural practices in the sites/neighborhoods of the study area.

The enrichment factors for the element, which were obtained using Mn as reference element and the elemental concentrations of reference crust (Loska et al. 1997). According to Table 2 the enrichment of the potential toxic elements like Cl and Pb could not be calculated because of the absence of these elements in the reference material (the control). Potassium, Ti and Mn showed moderate enrichment with ranged factor of (2–5) while Si, Ca and were significantly enriched with the ranged factor of (5–20). Iron showed the highest enrichment (Table 2) in this study having the average enrichment factor of 23.9. Obiajunwa et al. (2002) had advocated that enrichment factor greater than 10 can cause adverse health effect to soil plant, man and animal. Nevertheless the presence of Cl and Pb in the site of the study and the absence of the same in the control can be attributable to the pollution (contamination) from the burnt hospital waste.

**Table 2:** **Enrichment factor of elements in soil sampled around burnt hospital waste dump**

ELEMENTS	ENRICHMENT FACTOR			
	Site 1	Site 2	Site 3	Site 4
Si	7.2	6.8	7.6	7.9
Cl	ND	ND	ND	ND
K	1.9	2.5	2.4	2.7
Ca	14.7	13.6	13.1	12.4
Ti	5.1	5.8	5.6	4.6
V	ND	ND	ND	ND
Cr	3.7	2.9	3.2	3.1
Mn	1	1	1	1
Fe	24.7	27.0	25.3	23.6
Zr	13.3	14.5	15.2	12.2
Pb	ND	ND	ND	ND

*ND* = below detection limit.

The accuracy of our experimental values was assured by the analysis of the soil standard IAEA soil-7. The results of this analysis, presented in Table 3, are in good agreement with the certified values (IAEA 2000).

**Table 3:** **Results of Standard Reference Material**

ELEMENTS	IAEA SOIL 7 PIXE VALUE (ppm) IN THIS WORK	IAEA SOIL 7 CERTIFIED VALUE (ppm)
Si	144193.5±13309.06	144000
K	9697.1±101.71	9680
Ca	130333.1±208.53	130400
Ti	2400.9±31.45	2400
V	48.7±21.46	52.8
Cr	34.1 ± 11.68	48.0
Mn	531.3±17.80	504.8
Fe	20547.6±88.35	20560
Cu	19.4 ± 7.20	8.8
Zn	83.2 ± 9.88	83.2
Sr	86.5± 23.00	86.4
Zr	83.2±9.88	148
Pb	48.0±14.82	48.0

## Conclusion

We have filled the gap about the possible effect of the burnt hospital waste on agricultural soil. The PIXE technique used in the analysis of soil sampled around burnt hospital waste dump at the OAUTH Complex Ile-Ife, south western Nigeria was adequate for this kind of study. Eleven elements- Si, Cl, K, Ca, Ti, V, Cr, Mn, Fe, Zr and Pb were detected at an elevated concentration when compared with the control. In all highest enrichment was obtained in Fe. Moderate enrichment factors for Si, K, Ca, Ti, Cr and Zr were obtained. The level and the fate of these elements especially Cl and Pb is of serious environmental and health concern owing to the fact that there are intensive subsistence agricultural practices at and near the sites of the study. A future investigation to quantify dioxin and furan that is associated with the geochemistry of Cl is essential owing to the toxicity of these compounds.
